# Impact of life stage-dependent dispersal on the colonization dynamics of host patches by ticks and tick-borne infectious agents

**DOI:** 10.1186/s13071-017-2261-y

**Published:** 2017-08-04

**Authors:** Sarah Kada, Karen D. McCoy, Thierry Boulinier

**Affiliations:** 1 Centre d’Ecologie Fonctionnelle et Evolutive (CEFE) - CNRS Université Montpellier UMR 5175, 1919 route de Mende, 34293 Montpellier, France; 2 Maladies Infectieuses et Vecteurs: Ecologie, Génétique, Evolution et Contrôle, UMR CNRS 5290 - UR IRD 224 - Université Montpellier, Centre IRD, 34394 Montpellier, France

**Keywords:** Allee effect, *Borrelia burgdorferi*, Climate change, *Ixodes uriae*, Lyme disease, *Ornithodoros maritimus*, Parasite spread, Range expansion, Vertical transmission

## Abstract

**Background:**

When colonization and gene flow depend on host-mediated dispersal, a key factor affecting vector dispersal potential is the time spent on the host for the blood meal, a characteristic that can vary strongly among life history stages. Using a 2-patch vector-pathogen population model and seabird ticks as biological examples, we explore how vector colonization rates and the spread of infectious agents may be shaped by life stage-dependent dispersal. We contrast hard (Ixodidae) and soft (Argasidae) tick systems, which differ strongly in blood- feeding traits.

**Results:**

We find that vector life history characteristics (i.e. length of blood meal) and demographic constraints (Allee effects) condition the colonization potential of ticks; hard ticks, which take a single, long blood meal per life stage, should have much higher colonization rates than soft ticks, which take repeated short meals. Moreover, this dispersal potential has direct consequences for the spread of vector-borne infectious agents, in particular when transmission is transovarial.

**Conclusions:**

These results have clear implications for predicting the dynamics of vector and disease spread in the context of large-scale environmental change. The findings highlight the need to include life-stage dispersal in models that aim to predict species and disease distributions, and provide testable predictions related to the population genetic structure of vectors and pathogens along expansion fronts.

**Electronic supplementary material:**

The online version of this article (doi:10.1186/s13071-017-2261-y) contains supplementary material, which is available to authorized users.

## Background

Vector-borne disease emergence depends on the presence or spread of both a suitable vector and the associated infectious agent [1–7]. A growing number of models are used to describe temporal change in disease distributions [8] and to establish predictions on the risk of disease spread under climate change scenarios [9–15]. While climate is a key driver of vector range expansion because of its effects on habitat suitability [16–22], population structure and dispersal processes are also expected to play crucial roles in the speed of species’ spread [23]. Parasite dispersal is also an important driver of host-parasite coevolution [24], affecting local adaption [25, 26] and host and parasite genetic structure (e.g. Blouin et al. [27]; McCoy et al. [28]). The dynamics of dispersal, its speed and frequency, are particularly relevant for assessing vector expansion and the associated risk of spread of vector-borne infectious agents [12, 27, 29–31], but the key potential role of the dispersal stage is rarely considered in vector-borne disease models. Heterogeneity in dispersal rates among vector subpopulations is known to affect population genetic structure [28, 32, 33] and the geographical distribution of vector-borne infectious agents [33, 34]. For example, vector stage structure is thought to influence the speed and distance of vector colonization in ticks responsible for Lyme borreliosis [22, 35–37]. Dispersal distance in these ectoparasites may vary because of stage-specific host feeding preferences (e.g. nymphs feeding on birds versus small mammals; Wilson et al. [38]; Norte et al. [39]). Likewise, some stages might be more successful than others at (i) establishing a local population; and (ii) contributing to the spread of vector-borne infectious agents. For instance, releasing adults capable of breeding right away is considered to be more efficient than releasing non-reproductive juveniles in reintroduction programs [40, 41]. Also, density-dependent factors, notably Allee effects [30, 42, 43], can be related to difficulties for some stages to locate a mate or survive at low population densities [43, 44]. Allee effects are especially significant when studying the dynamics of population range expansion [45–47] because, by definition, the size of a newly established population is low. In addition, the maintenance of the infectious state across vector life stages (i.e. transstadial transmission, Randolph [48]; Hasle [21]) and vertical transmission to offspring (i.e. transmission from an infected adult female vector to its eggs, Rollend et al. [49]) will play critical roles in the emergence of vector-borne infectious agents [21, 48, 50, 51], and may interact with stage-dependent vector dispersal. Considering the impact of stage-dependent dispersal using a clear conceptual framework could therefore be particularly useful in order to gain a better understanding of the sources of eco-epidemiological variability in the context of vector and pathogen population expansions.

The ecological peculiarities of hard and soft ticks, i.e. the two main tick families, offer a unique opportunity to understand how stage-dependent dispersal strategies affect the process of tick colonization and the spread of tick-borne infectious agents. Tick dispersal is typically mediated by tick attachment on a vertebrate host for the blood meal [52, 53], and the two families have evolved contrasting feeding strategies [48, 54–56]. All hard tick life stages tend to have a long attachment duration (several days) on the hosts. Longer blood meal duration is generally associated with higher chances that the host moves to a new location, thereby increasing tick dispersal opportunities [57]. In contrast, soft ticks (particularly nymphs and adults) generally take several very short meals (15 to 30 min), mostly at night when the host is resting [54, 56, 58–60]. Dispersal opportunities for soft ticks may thus be more associated with the larval blood meal, as this meal may last several hours to days [61, 62]. These differences may have an effect on the relative capacities of hard and soft ticks (and their pathogens) to disperse and colonize new habitats [54, 63–65], and can be particularly important to consider in the current context of tick range expansions [6, 12, 66].

Here, we explore how differential stage-dependent dispersal of tick vectors, in interaction with demographic effects, conditions the colonization of suitable habitats and the spread of associated microparasites. We use a 2-patch modeling approach of a vector population structured in three explicit life stages (larva, nymph and adult), where one vector population may colonize the empty patch. We base our model on populations of soft and hard ticks that differ ecologically in their dispersal stage, and consider seabird-tick systems as case studies. Our hypothesis is that species for which only larval stages disperse, as is the case for soft ticks, will experience a time lag in population growth on a colonized patch because of (i) the time required to reach reproductive maturity, and (ii) potential Allee effects due to low dispersal rates. We expect these differences to affect the probability of successful vector colonization, but also the spread of vector-borne infectious agents among host patches. To explore this second aspect, we develop a Susceptible-Infected (SI, i.e. with no recovery) model of vector-borne infection for the vector population and SIR (Susceptible-Infected-Recovered) model for the vertebrate host, encompassing the relative contribution of stage-dependent vector dispersal. This allows us to evaluate the relative risks of spatial spread for microorganisms that use hard versus soft ticks as vectors.

### Why model seabird-tick systems?

**Fig. 1:**
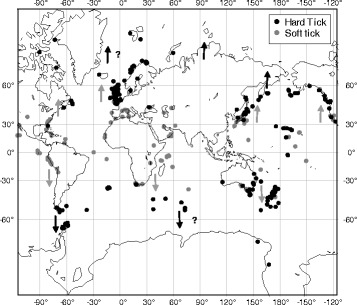
The known worldwide distribution of hard (*black dots*) and soft (*grey dots*) ticks of seabirds. Only ticks from the Ixodes and Ornithodoros (Carios) genera are represented. Arrows are illustrative and represent the potential colonization areas for hard (*black arrow*) and soft (*grey arrow*) seabird ticks, respectively. Data from Dietrich et al. [65]

Seabirds breed in discrete colonies that are highly structured in space (Furness & Monaghan [67]), providing dense and reliable sources of food and habitat for nest-dwelling ectoparasites like ticks [65]. These systems are particularly suitable for addressing questions related to tick dispersal because they involve a limited number of host species, notably compared to forest systems [12], and all hosts share common life history traits. Although only a limited number of tick species exploit seabirds, these ticks are present in most colonies and include species of the two main tick families, the Ixodidae and the Argasidae [65]. In seabird-tick systems, tick dispersal is passive, conditioned by host movements between breeding colonies during the breeding season [68–70] and the characteristics of the blood meal taken on the host. Dramatic differences exist between hard and soft ticks in terms of the length of the blood meal as outlined above. Finally, seabird colonies and their associated ticks are distributed at broad geographical scales [65, 71, 72], and seabird ticks are vectors of a broad range of viruses and bacteria [65], which make them especially interesting to study in the current context of climate change. Indeed, recent studies have revealed a northward expansion in the distribution of ixodid ticks [6, 66, 73]. Here, we consider two widespread seabird ticks, the hard tick *Ixodes uriae* which is found in the polar areas of both hemispheres, and the soft tick complex *Ornithodoros* (*Carios capensis*) (*sensu lato*) which lives in seabird colonies across the equatorial region [65]. As mean global temperatures increase, we may expect soft and hard tick populations to expand towards higher latitudes, where host populations exist for both species (see Fig. 1).

## Methods

### Population dynamics

In order to explore the potential effect of life-stage-dependent dispersal on vector colonization dynamics, we build a continuous-time model of vector population dynamics, structured in 3 life stages: larvae (*L*), nymphs (*Np*) and adults (*A*), on two patches (*i* and *j*). *σ*_*L*_ and *σ*_*N*_ are the maturation rates, i.e. the respective rates at which larvae moult to nymphs and nymphs to adults, provided they completed their blood meal. *μ*_*L*_, *μ*_*N*_ and *μ*_*A*_ are the respective natural mortality rates of larvae, nymphs and adults. The total population size is *N*_*v*_ = *L* + *Np* + *A*, and *m* ^*ij*^ and *m* ^*ji*^ represent dispersal rates between the two patches, which can differ among larvae, nymphs and adults (see below). The per capita birth rate of ticks is *b*_*v*_, and is scaled to a sex ratio of 0.5 and depends on the survival rate of eggs reaching the larval stage. The term $f\left({N}_v^i\right)$ represents the density-dependent growth of the total vector population in patch *i* and is dependent on the carrying capacity, *K*_*v*_ (density dependent constraint on tick population size assumed to be caused by the local availability of off-host shelters and the limit of tick density on a given host). We use the following expression:





The model is written:


For the sake of simplicity, and because we are mainly interested in the effect of stage-dependent dispersal, we consider all rates as continuous, even though vector activity follows a seasonal pattern under natural conditions [9, 74].

Tick dispersal occurs once the vector population reaches its equilibrium in the source patch. For the soft ticks, we consider that the blood meal is long enough for dispersal to occur only at the larval stage. Our assumption is that the propensity for dispersal is constrained by blood meal duration, therefore nymph and adult soft ticks, which take several short blood meals have no dispersal potential (*m*_*Np*_ = *m*_*A*_ = 0). For the hard ticks, the blood meal duration lasts several days across all stages. All stages are therefore considered to have the same probability of dispersing, such that we set equivalent dispersal rates: *m*_*L*_ = *m*_*Np*_ = *m*_*A*_ . Assuming a sex ratio of 0.5, only half of the adults have the opportunity to disperse, as only females take a blood meal after mating off-host with a male (Eveleigh & Threfall [75]). When comparing soft and hard tick dispersal towards an empty patch, we always let the same total number of individuals disperse. We use this approach because even though only larvae can disperse for soft ticks, these larvae can exploit the host throughout the reproductive season, whereas the different life stages of hard ticks tend to have specific, partially non-overlapping periods of host exploitation [76]. Moreover, we consider that this assumption results in a conservative comparison of the life-cycles.

### Allee effects on reproduction

Dispersing individuals at the edge of the distributional range can benefit from the low density of conspecifics because of a release from competition (positive density-dependent effect), but this low density may reduce population growth rate notably due to constraints on the probability of locating a mate (negative density-dependent effect, also known as Allee effect). Tick dispersal, which is achieved by host transportation, ensures arrival in a patch where hosts are available, but not necessarily where conspecifics are present at a high density. This can induce an Allee effect and result in a local decrease of the per capita birth rate, which could accordingly affect colonization success or time to vector establishment. To consider the impact of an Allee effect, the reproductive function was set to be dependent on the number of local adults. This constraint takes the following form:


where *a* is the strength of the Allee effect. An increase of *a* results in a decrease in local reproduction due to an increasing difficulty in locating a mate in the adult stage (see Additional file 1: Figure S1).

### Infection dynamics

**Fig. 2:**
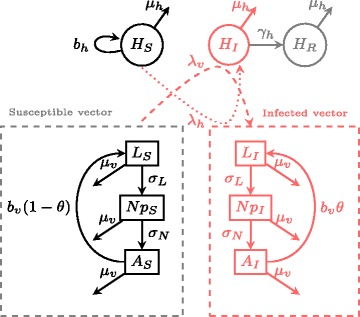
Schematic representation of the vector-borne infection model. Susceptible vectors from any stage (L_S_, Np_S_, A_S_) can become infected at rate **λ**_**v**_ when they feed on infected hosts (H_I_). Susceptible hosts can become infected at rate **λ**_h_ following a feeding event by an infected vector. Contact rate is set to 1 year^-1^ to respect the fact that ticks feed once per year. Equations are found in text

To explore the potential effect of life-stage dependent dispersal on the spread of vector-borne infectious agents, we then added the epidemiological dynamics of an infectious agent to our vector population model [77]. The vector population was set to follow a susceptible-infected (SI) model (see Fig. 2): larvae (*L*), nymphs (*Np*) and adults (*A*) are either susceptible (*S*) or infectious (*I*). We assume vectors do not recover from infection (i.e. no R compartment) and that its vital rates (birth, death and maturation) are unaltered (see Fig. 2). We also introduce infectious disease dynamics for the vertebrate host (SIR), as the infection is transmitted from an infected vector to a susceptible host (${H}_S^i$) and from an infected host (${H}_I^i$) to a susceptible vector. The 2-patch model describing the epidemiological dynamics in the vector population, for *i* = 1 , 2 is thus as follows:

And for the vertebrate host:


The host dynamics follow a classical SIR model, and the host density is determined by the local carrying capacity (*K*_*h*_). Transmission is frequency-dependent [78] and consequently depends on the proportion of infect hosts (*H*_*I*_) and vectors (*L*_*I*_, *Np*_*I*_, *A*_*I*_) in the populations. Transmission efficiency from host to vector and from vector to host is considered to be the same (*β*_*hv*_ = *β*_*vh*_), and vector competence is equivalent among life stages. Hosts can recover from the infection at rate γ_h_ *.* Infections in the vectors (${\lambda}_v^i$) and the hosts (${\lambda}_h^i$) are produced at rates:


Vertical transmission of the infection can occur between an infected adult female and a larva at rate *θ*. We do not consider direct, non-systemic transmission for this model (i.e. tick to tick transmission through co-feeding). Because survival between molts is high for tick-borne pathogens (e.g. *Borrelia burgdorferi* (*s*.*l*.), Bellet-Edimo et al. [79], Mitzel et al. [80]), transmission occurs transstadialy, meaning that infected larvae become infected nymphs. As ticks must take one blood meal per life stage, we assume one transmission event can occur per developmental stage. This is translated by a vector-host contact rate, *c*, set to 1 (e.g. Cobbold et al. [81]). By later varying *c* (see Additional file 2: Table S1), we investigate the impact of considering the multiple short blood meals that soft ticks take on the spread of infections. Although repeated blood meals at nymphal and adult stages could affect transmission among hosts within a patch, this will not lead to higher tick dispersal because, as discussed above, these blood meals are extremely short and taken at night.

For the sake of simplicity, the vector and vertebrate host populations are assumed to have reached their respective equilibrium in both patches (see Caraco et al. [35]). Additionally, we consider that the infection has reached its endemic equilibrium in the source patch before dispersal is introduced (in practical terms, *t* > 100 years). We then allow for dispersal between the two patches and investigate the effect of stage-dependent dispersal on the spread of infection. The term for dispersal, similar to that of Moore et al. [82], is as follows,


In this case, *m* ^*ij*^ represents the migration rate of the vector (*V*) from patch *i* to patch *j*. A vector can belong to the susceptible or infected class (*Y* ∈ {*S*, *I*}). Note that throughout the analyses, soft ticks disperse only at the larval stage whereas hard ticks are able to disperse at any stage. However, only half of the hard tick adults are able to disperse, as only females take a blood meal on the host. For the sake of simplicity, we only consider vector dispersal for the spread of the infectious agent, and not vertebrate host dispersal, because we are specifically interested in the contribution of the vector to the spread of vector-borne diseases.

To study the combined effects of stage-dependent dispersal and vertical transmission efficiency, we introduce a parameter for vertical (or transovarial) transmission, *θ*, a significant route of transmission for certain vector-borne infections [79, 83]. When *θ* = 0, there is no vertical transmission and all larvae are born susceptible. If *θ* > 0, then a proportion of eggs from an infected mother are born infected.

### Sensitivity analysis

In order to explore the robustness of results and their sensitivity to particular parameters, we performed a global sensitivity analysis [84]. The Latin Hypercube Sampling (LHS) method, which enables one to explore a wide range of parameter combinations, was performed using R 3.0.2 (*pse* package). Model outputs were obtained using Matlab 2010b for a range of parameter conditions (see Additional file 2: Table S1). The number of simulation runs (*N* = 150) respects the *N* > (4/3) *K* ratio for a sufficient level of relevance, *N* being the number of bootstrap replicates and *K* the number of unknown parameters. We let the population reach an equilibrium in the first patch (100 years) before allowing individuals to disperse to the second patch, and let the population and epidemiological models run for respectively 30 and 20 years, using the sampled parameter values. Model outputs were then analyzed using the Partial Rank Correlation Coefficient method (PRCC, R package *pse*, [85]) to determine which parameters had a large influence on colonization dynamics and the spread of vector-borne infections.

## Results

### Population dynamics and Allee effects

**Fig. 3:**
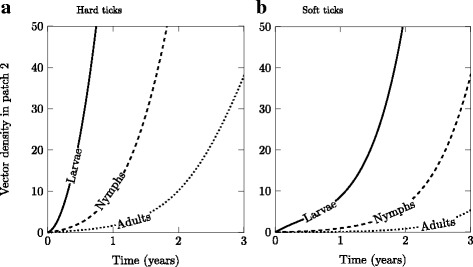
Density of **a** hard and **b** soft ticks in each life stage in the receiving patch. Default parameter values are found in Table 1. m_L_ = m_N_ = m_A_ = 0.01 year^-1^ for hard ticks, and the migration rate for soft ticks is adjusted so that an equivalent number of larval ticks disperse

For a broad set of reasonable parameter values (see Table 1), soft tick population growth in the newly colonized patch is much slower compared to the invading population of hard ticks (Fig. 3a, b). This can induce a time lag to establishment of a local population (i.e. time at which a viable number of individuals in the population is reached) of a local population which reflects a strong difference between the two tick types in the shape of population growth in the colonized patch. Soft ticks experience a delay in the local production of offspring, thus in local population growth, because of the required developmental time and associated likelihood for larvae to reach adulthood (Fig. 3b). The population dynamics observed at the early stage of invasion by soft ticks highlights the essential role of immigrants from the source patch (i.e. *propagule rain*, Gotelli [86]) in initiating local reproduction. For the sake of simplicity, our model assumed the same number of dispersing individuals for hard and soft ticks by using a higher dispersal rate for larvae of soft ticks. Although potentially justified based on the phenological differences in host exploitation between soft and hard ticks (see above), this advantage may be overestimated and soft ticks may experience even lower rates of colonization than suggested in our model.

**Table 1:** Parameters used for the population dynamics and epidemiological models

Parameter	Description	Value (.year^-1^)
Vector		
*b*_*v*_	Number of eggs per adult female tick surviving to reach larval stage	200
*K*_*v*_	Carrying capacity for vector	1e3
*μ*_*L*_	Mortality rate per tick larva	0.95
*μ*_*N*_	Mortality rate per tick nymph	0.3
*μ*_*A*_	Mortality rate per adult tick	0.4
*σ*_*L*_	Maturation rate from larvae to nymph	0.3
*σ*_*N*_	Maturation rate from nymph to adult	0.3
*m*_*ij*_	Migration rate	variable (from 0.01 to 0.02)
*α*	Strength of the Allee effect	variable (from 1 to 40)
Vertebrate host		
*b*_*h*_	Vertebrate host birth rate	1.4 per pair
*K*_*h*_	Carrying capacity for the vertebrate host	3e2
*μ*_*h*_	Vertebrate host mortality rate	0.12
Epidemiological parameters		
*β*_*hv*_	Transmission from tick to vertebrate host	0.8
*β*_*vh*_	Transmission from vertebrate host to tick	0.5
*γ*_*h*_	Average duration of infection	0.33
*θ*	Vertical transmission from adult tick to eggs	variable (from 0.001 to 0.1)
*c*	Yearly contact rate (number of yearly bloodmeal)	variable (from 1 to 1.2)

**Fig. 4:**
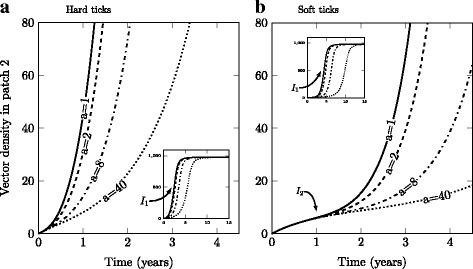
Strength of the Allee effect and total vector population density in the receiving patch for **a** hard and **b** soft ticks (a = 1, 2, 8 and 40; higher values indicate a stronger Allee effect). The arrows are for illustrative purposes. They represent the inflexion points for the short (I_2_) and long-term (I_1_) dynamics. On the right panel (**b**), the inflexion point (I_1_), represents the threshold above which the population growth becomes positive. Default parameter values are found in Table 1. m_L_ = 0.017 year^-1^ for the soft and m_L_ = m_N_ = m_A_ = 0.01 year^-1^ for hard ticks

Including an Allee effect on vector populations in our model created an even greater disparity between hard and soft tick colonization dynamics. When population size is low, the Allee effects (affecting local reproduction) limit the population growth for only a very short period for the hard tick (Fig. 4a), but lasts over a much longer period for the soft tick (Fig. 4b). Here again, this is easily understood because the dispersal of only juvenile tick life stages delays the local buildup of a breeding population. As in the case with no Allee effect, the soft tick population growth exhibits a different shape than the expected logistic curve we find for the hard tick population. We see a clear point of inflexion (I_2_) at the beginning of the soft tick population growth curve, after an initial concave period (Fig. 4b). This concavity again reflects the propagule rain in the absence of local reproduction. Once a threshold number of mature adults is attained, the population growth then follows a logistic curve.

### Infection dynamics

**Fig. 5:**
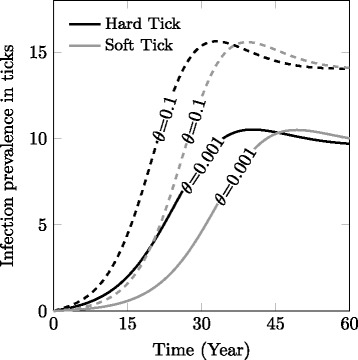
Infection spread in the new patch through stage-dependent and bidirectional dispersal prevalence for low (*solid lines*, θ = 0.001) and high vertical (transovarial) transmission rates (θ = 0.1, *dashed lines*), following arrival of susceptible and infected ticks from patch 1. Default parameters are found in Table 1. Only 1 bloodmeal per stage (c = 1) is considered. The polyphagous nature of soft ticks (c > 1) partially compensates for the lag in local density, but the infection prevalence in soft ticks, still does not reach that of hard ticks (see Additional file 2: Figure S2, for details)

In this model, an epidemiological component is integrated into the previous tick dispersal model. We find that infection prevalence in the infection-free patch increases faster in the hard tick population than in the soft tick population (Fig. 5). Dispersing nymphs and adults are associated with a greater transmission risk compared to dispersing larvae because of their higher survival rate. Higher prevalence is found when vertical transmission is high (see Fig. 5, dashed line), and increases significantly when hard ticks disperse because the arrival of adult ticks directly translates into a higher number of infected individuals. The repeated blood meals in the soft tick life-cycle also result in higher infection prevalence due to increased contact rates with the host (Additional file 2: Figure S2), but never equals diffusion rates in hard ticks. Because soft ticks are nidicolous, repeated blood meals may be taken on the same host individual such that the potential increase in infection prevalence due to this life history trait may be overestimated by a model not accounting for local spatial structure.

### Sensitivity analysis

**Fig. 6:**
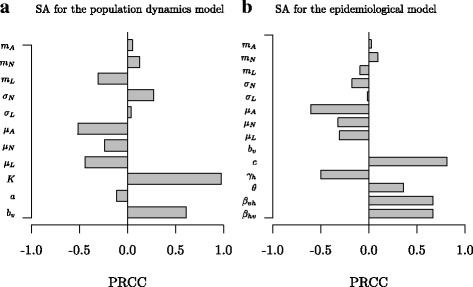
Results of the sensitivity analysis (SA) for the vector population size in the newly colonized patch after 30 years (**a**) and the prevalence of the infection in this patch after 30 years (**b**), using the PRCC index which indicates the quantitative impact and direction of the relationship between a parameter and the model output. The SA is performed for 150 runs of the LHS matrix. Parameter used for the models are found in Additional file 2: Table S1

The results of the LHS-PRCC sensitivity analysis indicate that vector birth rate (*b*_*v*_), carrying capacity (*K*) and nymphal maturation rate (*σ*_*N*_) have a strong positive impact on vector population density on patch 2. Nymphal and adult dispersal rates also have a positive relationship with vector population density, while larval dispersal, the strength of the Allee effect and mortality rates of all tick stages negatively interact with it (Fig. 6a). As expected for the epidemiological model, the PRCC index showed a strong positive relationship of transmission rates (systemic, *β*_*vh*_, *β*_*hv*_ and vertical, *θ* transmission), contact rate between hosts and vector (*c*) on the infection prevalence in patch 2, while the vertebrate host recovery rate (_*γh*_) negatively influences infection prevalence (Fig. 6b). Dispersal rates do not strongly affect the prevalence of infection, but interestingly we remark that nymphal and adult dispersal rates positively influenced infection prevalence, while larval dispersal negatively influenced it.

## Discussion

### Population dynamics

Parasite dispersal is a key component of host-parasite ecology and coevolution [24]. One of the key challenges in the eco-epidemiology of vector-borne diseases is to understand the dynamics that may explain vector species distributions at a hierarchy of temporal scales. While climate change has an inevitable impact on the establishment of vector species and the spread of vector-borne diseases at different spatial scales [87], the processes governing dispersal capacity have been largely overlooked in the case of vector species with complex life-cycles, such as ticks (but see Auld & Tinsley [88]). Vector populations are a striking example of stage-structured populations where life-stage peculiarities may feedback on ecological and epidemiological dynamics [30, 35, 89], and might also impact the genetic structure of vector species and their infectious agents. Our results show that stage-dependent dispersal, in association with maturation time and the survival probabilities of immature stages, is likely to constrain strongly the time-to-establishment of soft tick populations. The differences in population growth and establishment success we observe for hard and soft ticks are produced by the contrasted dispersal opportunities and their demographic effects. The general implications of our results suggest that inclusion of differential dispersal opportunities among life-stages could help predict the expansion of vector species more accurately. Specifically, we may expect strong differences in the speed of colonization for soft and hard ticks at comparable spatial scales. While we did not explicitly allow for dispersal rates to depend on blood meal lengths, it is likely tightly linked to dispersal opportunity. For instance, Heylen & Matthysen [57] suggested that the contrasted feeding duration and detachment timing (day versus night) between two ixodid ticks feeding on the same songbird lead to strong differences in their respective dispersal capacities. Further empirical work is now needed to understand how blood meal length and detachment strategies influence tick dispersal potential.

We also found that density-dependent mechanisms (Allee effects, notably related to the difficulty in finding mates at low density) are likely to interact with stage-dependent dispersal to strongly affect colonization dynamics. This effect is particularly strong for soft ticks, for which we observed strongly delayed population growth at the early stages of invasion compared to hard ticks. Establishment success typically depends on the local immigration of particular life-stages, notably adults, due to their rapid contribution to local reproduction.

### Infection dynamics

Predicting the speed of vector-borne disease spread depends on our ability to understand eco-epidemiological peculiarities of the system. Our model shows that stage-dependent dispersal and particular epidemiological parameters can play critical roles in the spread of vector-borne infections. Given the importance of stage structure for vector-borne disease dynamics in relation to host use and tick stage susceptibility [90], a better understanding such eco-evolutionary processes should result in a better appraisal of the conditions favoring the spread of vector-borne infections (e.g. Pelosse et al. [30]). We showed here, using a simple model, that stage at dispersal and vertical transmission play an important role on the spread of vector-borne infections.

Although our results show that hard tick vectors should spread infectious agents more rapidly than soft ticks, the detailed biological cycle of soft ticks may temper our views regarding their ability to spread vector-borne infections. Soft ticks have many nymphal instars, each typically requiring at least one blood meal, and adult ticks feed numerous times. This repeated contact with the host can potentially increase the transmission of vector-borne infectious agents, and can lead to a faster increase in infection prevalence (see Additional file 2: Figure S2). However, the spread of infection in soft tick systems and our ability to compare predictions to other vector types will depend on the specific ecology of the tick species and whether or not it feeds on different host individuals at each blood meal. Indeed, the nidicolous lifestyle of many soft ticks may limit the transmission of infectious agents because ticks are restricted to hosts found in the same nest, or burrow. As mentioned in the model description, it is unlikely that the multiple blood meals per stage of soft ticks will increase their dispersal capacity (and thus colonization rate), because these meals are of very short duration (few minutes) and tend to be taken while the host is sedentary. The spatial restriction in the distribution of infectious agents found in examined soft tick species to date is consistent with these assumptions and model predictions. For instance, Arnal et al. [91] screened 19 Yellow-legged gull (*Larus michahellis*) colonies in the Mediterranean Sea for the local circulation of a Meaban-like virus, both by testing for specific antibodies in egg yolks, and by directly detecting viral RNA in the soft tick vector, *Ornithodoros maritimus*. They observed the presence of the tick in many of the colonies, but over the 3-year observation period only found one colony positive for the presence of the virus. They explained this restricted distribution by the high fidelity of gulls to their nesting sites combined with the limited dispersal abilities of the soft tick. A recent study found a similar restricted pattern of presence of the Meaban-like virus in other host species, further supporting the hypothesis of a limited dispersal capacity of soft ticks [92]. In this case, and more generally, we can expect tick dispersal characteristics to affect the timing of tick-borne infection establishment, and thus this trait needs to be incorporated directly into predictive models of disease spread. This should notably be important for species distribution models that may be proposed to predict range shifts in hosts and parasites, and their potential consequences for biodiversity.

### Implications for epidemiology and population genetic structure

The results presented here have several implications for the genetic structure of ticks and tick-borne infectious agents. Because of their contrasted stage-dependent feeding behaviors, soft ticks may experience lower colonization success and restricted gene flow among populations. For the infectious agents that rely on these vectors, their genetic diversity and population structure may be affected in cascade depending on their mode of transmission [93]. To our knowledge, no study has yet examined the population genetic structure of soft ticks using hypervariable markers, but high diversity and strong clade structure at conserved genes was found in *Ornithodoros sonrai* (Argasidae), a soft tick living in rodent burrows in west Africa, suggesting relatively low dispersal rates [94]. Based on our model results, we generally expect higher population genetic structure to be found for soft tick species than for hard ticks and that this structure may contribute to reducing the circulation of the vector-borne infectious agents. Molecular studies of infectious agents shared by both soft and hard ticks would be ideal to test these predictions [95, 96]. In this context, seabird ticks interacting with various flaviviruses and Borrelia bacteria (e.g. Lyme disease versus relapsing fever) could represent worthwhile systems to explore [97, 98].

### Model limitations

The present model relies on several simplifications: (i) the life-cycles of soft and hard tick vectors (i.e. similar maturation rates and cycle), (ii) assumption that the source population is at equilibrium, (iii) absence of seasonality. We expect these simplifications to either be unimportant, or to result in more extreme differences between the vector groups. In this way, our basic model contributes substantially to a better understanding of how patterns of tick dispersal influence species spread (e.g. Leighton et al. [20]). For simplicity, and to better track the impact of tick movements, our model only accounts for vector movements for the introduction of the vector-borne infections. Host movements influence vector dispersal rates, and can also be the source of local infections when an infected individual disperses to a new patch where a vector population is already present. Migratory birds are, for instance, hypothesized to be responsible for the introduction of a number of such infectious agents (e.g. West Nile virus introduction in the New World; Rappole & Hubálek [99]; Gubler [100]). Recent population genetics studies have allowed us to better track vector movements between populations [28, 101], but require further developments to inform us about the relative contribution of vector *vs* vertebrate host to vector-borne parasite structure. Finally, our model does not take into account the alternative transmission mode of the pathogen via co-feeding, where an infected tick transmits the infectious agent directly to other ticks feeding on the host, without a systemic infection in the host itself. We would expect co-feeding to similarly impact hard and soft tick systems, such that our general results and predictions would not change.

## Conclusions

Using our model, we highlight the importance of considering the ecological properties of the vector life-cycle, notably the role of stage-dependent dispersal, for understanding colonization potential and gene flow in the vector and in associated parasites. Although this model is based on seabird-tick systems, it is conceptually valid to be generalized to other vector-borne systems. As several tick species are currently expanding their geographic distributions in relation to global changes [6], our approach may be especially useful to assess the associated invasion risk of vector-borne pathogens. Other ecological factors, such as sex-biased vector dispersal [102] or host-associated dispersal in multi-host systems [28], would also be worthwhile to consider with this framework to better understand and predict the spatial spread of vector-borne infections.

## Additional files

Additional file 1: Figure S1. Density-dependence function (f) with respect to varying strength of the Allee effect (a). Where K = 600, 200, 100 and a vary from 0 to 100. High a reduces the population reproductive function and represents notably the difficulty to locate a mate when the population density is low. (PDF 48 kb)

Additional file 2: To fully understand the consequences of the soft tick polyphasic cycle on vector-infection spread, we investigated the impact of an increase in the parameter feeding rate, c. This contact rate is only increased for nymphal and adult stages, as soft ticks take multiple short bloodmeals during those stages, while they only feed once during the larval stage, therefore c is kept equal to 1 at the larval stage. A slight increase in feeding rate leads to a rapid rise in the infection prevalence in soft tick systems, but as the soft ticks nidicolous lifestyle means that they probably feed on the same host or its offspring, soft tick multiple bloodmeal per life stage would certainly not increase vector-borne infection prevalence to the extent induced by direct changes of c. **Figure S2.** Vector-borne infection prevalence for different contact rates (i.e. tick feeding rate). Hard tick feed only once a year (c = 1 at all times), while soft can feed multiple times during nymphal and adult stages (c > 1 for nymphs and adults, c = 1 for larvae), which could potentially increase the opportunity for transmission, if they are to feed on different hosts. Default parameters are found in Table 1, θ = 0.001. **Table S1.** Parameters used for sensitivity analysis. (ZIP 217 kb)

## References

[1] Purse BV, Mellor PS, Rogers DJ, Samuel AR, Mertens PPC, Baylis M (2005). Climate change and the recent emergence of bluetongue in Europe. Nat Rev Microbiol.

[2] Benedict MQ, Levine RS, Hawley WA, Lounibos LP (2007). Spread of the tiger: global risk of invasion by the mosquito *Aedes albopictus*. Vector-Borne Zoonotic Dis..

[3] Lemon SM, Sparling PF, Hamburg MA, Relman DA, Choffnes ER, Mack A (2008). Vector-borne diseases: understanding the environmental, human health, and ecological connections, workshop summary.

[4] Lafferty KD (2009). The ecology of climate change and infectious diseases. Ecology.

[5] Mills JN, Gage KL, Khan AS (2010). Potential influence of climate change on vector-borne and zoonotic diseases: a review and proposed research plan. Environ Health Perspect.

[6] Léger E, Vourc’h G, Vial L, Chevillon C, McCoy KD (2013). Changing distributions of ticks: causes and consequences. Exp Appl Acarol..

[7] Van Hemert C, Pearce JM, Handel CM (2014). Wildlife health in a rapidly changing North: focus on avian disease. Front Ecol Environ.

[8] Estrada-Peña A, Ostfeld RS, Peterson AT, Poulin R, de la Fuente J (2014). Effects of environmental change on zoonotic disease risk: an ecological primer. Trends Parasitol.

[9] Randolph SE, Green RM, Peacey MF, Rogers DJ (2000). Seasonal synchrony: the key to tick-borne encephalitis foci identified by satellite data. Parasitology.

[10] Brownstein JS, Holford TR, Fish D (2005). Effect of climate change on Lyme disease risk in North America. EcoHealth.

[11] Ogden NH, Maarouf A, Barker IK, Bigras-Poulin M, Lindsay LR, Morshed MG (2006). Climate change and the potential for range expansion of the Lyme disease vector *Ixodes scapularis* in Canada. Int J Parasitol.

[12] Ogden NH, Mechai S, Margos G (2013). Changing geographic ranges of ticks and tick-borne pathogens: drivers, mechanisms and consequences for pathogen diversity. Front Cell Infect Microbiol.

[13] Loiseau C, Harrigan RJ, Cornel AJ, Guers SL, Dodge M, Marzec T (2012). First evidence and predictions of *Plasmodium* transmission in Alaskan bird populations. PLoS One.

[14] Simon JA, Marrotte RR, Desrosiers N, Fiset J, Gaitan J, Gonzalez A (2014). Climate change and habitat fragmentation drive the occurrence of *Borrelia burgdorferi*, the agent of Lyme disease, at the northeastern limit of its distribution. Evol Appl.

[15] Péron G, Altwegg R, Jamie GA, Spottiswoode CN (2016). Coupled range dynamics of brood parasites and their hosts responding to climate and vegetation changes. J Anim Ecol.

[16] Daszak P, Cunningham AA, Hyatt AD (2000). Emerging infectious diseases of wildlife - threats to biodiversity and human health. Science.

[17] Gray JS, Dautel H, Estrada-Peña A, Kahl O, Lindgren E (2009). Effects of climate change on ticks and tick-borne diseases in Europe. Interdiscip Perspect Infect Dis.

[18] Harvell D, Altizer S, Cattadori IM, Harrington L, Weil E (2009). Climate change and wildlife diseases: When does the host matter the most?. Ecology.

[19] Estrada-Peña A, Ayllón N, de la Fuente J (2012). Impact of climate trends on tick-borne pathogen transmission. Front Physiol.

[20] Leighton PA, Koffi JK, Pelcat Y, Lindsay R, Ogden NH (2012). Predicting the speed of tick invasion: an empirical model of range expansion for the Lyme disease vector *Ixodes scapularis* in Canada. J Anim Ecol.

[21] Hasle G (2013). Transport of ixodid ticks and tick-borne pathogens by migratory birds. Front Cell Infect Microbiol.

[22] Medlock JM, Hansford KM, Bormane A, Derdakova M, Estrada-Peña A, George J-C (2013). Driving forces for changes in geographical distribution of *Ixodes ricinus* ticks in Europe. Parasit Vectors.

[23] Neubert MG, Caswell H (2000). Demography and dispersal: calculation and sensitivity analysis of invasion speed for structured populations. Ecology.

[24] Thompson JN (1994). The Coevolutionary Process.

[25] Gandon S, Capowiez Y, Dubois Y, Michalakis Y, Olivieri I (1996). Local adaptation and gene-for-gene coevolution in a metapopulation model. Proc R Soc Lond B Biol Sci.

[26] Gandon S, Michalakis Y (2002). Local adaptation, evolutionary potential and host-parasite coevolution: interactions between migration, mutation, population size and generation time. J Evol Biol.

[27] Blouin MS, Yowell CA, Courtney CH, Dame JB (1995). Host movement and the genetic structure of populations of parasitic nematodes. Genetics.

[28] McCoy KD, Boulinier T, Tirard C, Michalakis Y (2003). Host-dependent genetic structure of parasite populations: differential dispersal of seabird tick host races. Evolution.

[29] Hartfield M, White KAJ, Kurtenbach K (2011). The role of deer in facilitating the spatial spread of the pathogen *Borrelia burgdorferi*. Theor Ecol.

[30] Pelosse P, Kribs-Zaleta CM, Ginoux M, Rabinovich JE, Gourbière S, Menu F (2013). Influence of vectors’ risk-spreading strategies and environmental stochasticity on the epidemiology and evolution of vector-borne diseases: the example of Chagas’ disease. PLoS One.

[31] Hall RJ, Brown LM, Altizer S. Modeling vector-borne disease risk in migratory animals under climate change. Integr Comp Biol. 2016;56:353-64. 10.1093/icb/icw049 27252225

[32] Frank SA (1986). Dispersal polymorphisms in subdivided populations. J Theor Biol.

[33] Rebaudo F, Costa J, Almeida CE, Silvain J-F, Harry M, Dangles O (2014). Simulating population genetics of pathogen vectors in changing landscapes: guidelines and application with *Triatoma brasiliensis*. PLoS Negl Trop Dis.

[34] Gatewood AG, Liebman KA, Vourc’h G, Bunikis J, Hamer SA, Cortinas R (2009). Climate and tick seasonality are predictors of *Borrelia burgdorferi* genotype distribution. Appl Environ Microbiol.

[35] Caraco T, Glavanakov S, Chen G, Flaherty JE, Ohsumi TK, Szymanski BK (2002). Stage-structured infection transmission and a spatial epidemic: a model for Lyme disease. Am Nat.

[36] Madhav NK, Browstein JS, Tsao JI, Fish D (2004). A dispersal model for the range expansion of blacklegged tick (Acari: Ixodidae). J Med Entomol.

[37] Ogden NH, Bigras-Poulin M, Hanincova K, O’Callaghan CJ, Kurtenbach K (2008). Projected effects of climate change on tick phenology and fitness of pathogens transmitted by the North American tick *Ixodes scapularis*. J Theor Biol.

[38] Wilson ML, Litwin TS, Gavin TA, Capkanis MC, Maclean DC, Spielman A (1990). Host-dependent differences in feeding and reproduction of *Ixodes dammini* (Acari: Ixodidae). J Med Entomol.

[39] Norte AC, de Carvalho IL, Ramos JA, Gonçalves M, Gern L, Núncio MS (2012). Diversity and seasonal patterns of ticks parasitizing wild birds in western Portugal. Exp Appl Acarol.

[40] Pearce-Kelly P, Jones R, Clarke D, Walker C, Atkin P, Cunningham AA (1998). The captive rearing of threatened Orthoptera: a comparison of the conservation potential and practical considerations of two species’ breeding programmes at the Zoological Society of London. J Insect Conserv.

[41] Sarrazin F, Legendre S (2000). Demographic approach to releasing adults *versus* young in reintroductions. Conserv Biol.

[42] Allee WC, Park O, Emerson AE, Park T, Schmidt KP (1949). Principles of animal ecology.

[43] Courchamp F, Clutton-Brock T, Grenfell B (1999). Inverse density dependence and the Allee effect. Trends Ecol Evol.

[44] Liebhold A, Bascompte J (2003). The Allee effect, stochastic dynamics and the eradication of alien species. Ecol Lett.

[45] Lewis MA, Kareiva P (1993). Allee dynamics and the spread of invading organisms. Theor Popul Biol.

[46] Keitt TH, Lewis Mark A, Holt RD (2001). Allee effects, invasion pinning, and species’ borders. Am Nat.

[47] Contarini M, Onufrieva KS, Thorpe KW, Raffa KF, Tobin PC (2009). Mate-finding failure as an important cause of Allee effects along the leading edge of an invading insect population. Entomol Exp Appl.

[48] Randolph SE (1998). Ticks are not insects: consequences of contrasting vector biology for transmission potential. Parasitol Today.

[49] Rollend L, Fish D, Childs JE (2013). Transovarial transmission of *Borrelia* spirochetes by *Ixodes scapularis*: a summary of the literature and recent observations. Ticks Tick-Borne Dis.

[50] Hartemink NA, Randolph SE, Davis SA, Heesterbeek JAP (2008). The basic reproduction number for complex disease systems: defining R_0_ for tick-borne infections. Am Nat.

[51] Nonaka E, Ebel GD, Wearing HJ (2010). Persistence of pathogens with short infectious periods in seasonal tick populations: the relative importance of three transmission routes. PLoS One.

[52] Falco RC, Fish D (1991). Horizontal movement of adult *Ixodes dammini* (Acari: Ixodidae) attracted to Co2-Baited Traps. J Med Entomol.

[53] Sonenshine DE, Mather TN (1994). Ecological dynamics of tick-borne zoonoses.

[54] Vial L (2009). Biological and ecological characteristics of soft ticks (Ixodida: Argasidae) and their impact for predicting tick and associated disease distribution. Parasite.

[55] Hajdušek O, Šíma R, Ayllón N, Jalovecká M, Perner J, de la Fuente J (2013). Interaction of the tick immune system with transmitted pathogens. Front Cell Infect Microbiol.

[56] Sonenshine DE, Roe RM (2013). Biology of ticks.

[57] Heylen DJA, Matthysen E (2010). Contrasting detachment strategies in two congeneric ticks (Ixodidae) parasitizing the same songbird. Parasitology.

[58] Baker JR, Muller R, Rollinson D. Preface. In: Baker JR, Muller R, Rollinson D, editors, Adv Parasitol. 2006;63:vii-ix. http://www.sciencedirect.com/science/article/pii/S0065308X05620179.

[59] Capinera JL (2008). Encyclopedia of Entomology.

[60] Ribeiro CCDU, Faccini JLH, Cançado PHD, Piranda EM, Barros-Battesti DM, Leite RC (2013). Life cycle of *Ornithodoros rostratus* (Acari: Argasidae) under experimental conditions and comments on the host-parasite relationship in the Pantanal wetland region. Brazil Exp Appl Acarol.

[61] Endris RG, Haslett TM, Monahan MJ, Phillips JG (1991). Laboratory biology of *Ornithodoros* (*Alectorobius*) *puertoricensis* (Acari: Argasidae). J Med Entomol.

[62] Dautel H, Knülle W (1997). The influence of physiological age of *Argas reflexus* larvae (Acari: Argasidae) and of temperature and photoperiod on induction and duration of diapause. Oecologia.

[63] Heath A. Zoogeography of the New Zealand tick fauna. Tuatara. 1977;23:26–39.

[64] Ravaomanana J, Michaud V, Jori F, Andriatsimahavandy A, Roger F, Albina E (2010). First detection of African swine fever virus in *Ornithodoros porcinus* in Madagascar and new insights into tick distribution and taxonomy. Parasit Vectors.

[65] Dietrich M, Gómez-Díaz E, McCoy KD (2011). Worldwide distribution and diversity of seabird ticks: implications for the ecology and epidemiology of tick-borne pathogens. Vector-Borne Zoonotic Dis.

[66] Jaenson TG, Jaenson DG, Eisen L, Petersson E, Lindgren E (2012). Changes in the geographical distribution and abundance of the tick *Ixodes ricinus* during the past 30 years in Sweden. Parasit Vectors.

[67] Furness RW, Monaghan P (1987). Seabird ecology.

[68] Danchin E (1992). The incidence of the tick parasite *Ixodes uriae* in Kittiwake *Rissa tridactyla* colonies in relation to the age of the colony, and a mechanism of infecting new colonies. Ibis.

[69] McCoy KD, Boulinier T, Tirard C (2005). Comparative host-parasite population structures: disentangling prospecting and dispersal in the black-legged kittiwake *Rissa tridactyla*. Mol Ecol.

[70] Boulinier T, Kada S, Dupraz M, Aurore Ponchon, Dietrich M, Gamble A, et al. Migration, prospecting, dispersal? What host movement matters for infectious agent circulation? Integr Comp Biol. 2016;56(2):330-42. 10.1093/icb/icw015 27252195

[71] Olsen B, Duffy DC, Jaenson TG, Gylfe A, Bonnedahl J, Bergström S (1995). Transhemispheric exchange of Lyme disease spirochetes by seabirds. J Clin Microbiol.

[72] Dietrich M, Kempf F, Boulinier T, McCoy KD (2014). Tracing the colonization and diversification of the worldwide seabird ectoparasite *Ixodes uriae*. Mol Ecol.

[73] Estrada-Peña A, Sánchez N, Estrada-Sánchez A (2012). An assessment of the distribution and spread of the tick *Hyalomma marginatum* in the western Palearctic under different climate scenarios. Vector Borne Zoonotic Dis.

[74] Ogden NH, Bigras-Poulin M, O’Callaghan CJ, Barker IK, Kurtenbach K, Lindsay LR (2007). Vector seasonality, host infection dynamics and fitness of pathogens transmitted by the tick *Ixodes scapularis*. Parasitology.

[75] Eveleigh ES, Threfall W. The biology of *Ixodes* (*Ceratixodes*) *uriae* White, 1852 in Newfoundland. Acarologia. 1974;16:621–53. 1179956

[76] Barton TR, Harris MP, Wanless S, Elston DA (1996). The activity periods and life-cycle of the tick *Ixodes uriae* (Acari: Ixodidae) in relation to host breeding strategies. Parasitology.

[77] Keeling MJ, Rohani P. Modeling infectious diseases in humans and animals. Princeton: Princeton University Press; 2008 [cited 2015 Jun 8]. Available from: http://books.google.com/books?hl=en&lr=&id=G8enmS23c6YC&oi=fnd&pg=PP2&dq=info:gKeYdpqNt6UJ:scholar.google.com&ots=rFEPCv5opS&sig=UK32GGPw1ZSodHQywkRZexZy_YA

[78] Antonovics J, Iwasa Y, Hassell MP (1995). A generalized model of parasitoid, venereal, and vector-based transmission processes. Am Nat.

[79] Bellet-Edimo R, Betschart B, Gern L (2005). Frequency and efficiency of transovarial and subsequent transstadial transmission of *Borrelia burgdorferi sensu lato* in *Ixodes ricinus* ticks. Bull Société Neuchâtel Sci Nat.

[80] Mitzel DN, Wolfinbarger JB, Long RD, Masnick M, Best SM, Bloom ME (2007). Tick-borne flavivirus infection in *Ixodes scapularis* larvae: development of a novel method for synchronous viral infection of ticks. Virology.

[81] Cobbold CA, Teng J, Muldowney JS (2015). The influence of host competition and predation on tick densities and management implications. Theor Ecol.

[82] Moore SM, Manore CA, Bokil VA, Borer ET, Hosseini PR (2011). Spatiotemporal model of barley and cereal yellow dwarf virus transmission dynamics with seasonality and plant competition. Bull Math Biol.

[83] Danielová V, Holubová V, Dusbabek F, Bukva V (1991). Transovarial transmission rate of tick-borne encephalitis virus in *Ixodes ricinus* ticks. Modern acarology.

[84] Wu J, Dhingra R, Gambhir M, Remais JV (2013). Sensitivity analysis of infectious disease models: methods, advances and their application. J R Soc Interface.

[85] Chalom A, de Prado PIK. Parameter space exploration of ecological models. arXiv. 2012 [cited 2016 May 26]; Available from: http://arxiv.org/abs/1210.6278

[86] Gotelli NJ (1991). Metapopulation models: the rescue effect, the propagule rain, and the core-satellite hypothesis. Am Nat.

[87] Sutherst RW (1998). Implications of global change and climate variability for vector-borne diseases: generic approaches to impact assessments. Int J Parasitol.

[88] Auld SKJR, Tinsley MC (2015). The evolutionary ecology of complex life-cycle parasites: linking phenomena with mechanisms. Heredity.

[89] Boulinier T, McCoy K, Sorci G, Clobert J, Danchin E, Dhondt AA, Nichols JD (2001). Dispersal and parasitism. Dispersal.

[90] Klepac P, Pomeroy LW, Bjørnstad ON, Kuiken T, Osterhaus ADME, Rijks JM (2009). Stage-structured transmission of phocine distemper virus in the Dutch 2002 outbreak. Proc R Soc B Biol Sci.

[91] Arnal A, Gómez-Díaz E, Cerdà-Cuéllar M, Lecollinet S, Pearce-Duvet J, Busquets N, et al. Circulation of a Meaban-like virus in yellow-legged gulls and seabird ticks in the western Mediterranean Basin. PLoS One. 2014;9:e89601. 10.1371/journal.pone.0089601 PMC3953012 24625959

[92] Jurado-Tarifa E, Napp S, Lecollinet S, Arenas A, Beck C, Cerdà-Cuéllar M (2016). Monitoring of West Nile virus, Usutu virus and Meaban virus in waterfowl used as decoys and wild raptors in southern Spain. Comp Immunol Microbiol Infect Dis.

[93] Archie EA, Luikart G, Ezenwa VO (2009). Infecting epidemiology with genetics: a new frontier in disease ecology. Trends Ecol Evol.

[94] Vial L, Durand P, Arnathau C, Halos L, Diatta G, Trape JF (2006). Molecular divergences of the *Ornithodoros sonrai* soft tick species, a vector of human relapsing fever in West Africa. Microbes Infect Inst Pasteur.

[95] Biek R, Real LA (2010). The landscape genetics of infectious disease emergence and spread. Mol Ecol.

[96] Mazé-Guilmo E, Blanchet S, McCoy KD, Loot G (2016). Host dispersal as the driver of parasite genetic structure: a paradigm lost?. Ecol Lett.

[97] Heinze DM, Gould EA, Forrester NL (2012). Revisiting the clinical concept of evolution and dispersal for the tick-borne flaviviruses by using phylogenetic and biogeographic analyses. J Virol.

[98] McCoy KD, Léger E, Dietrich M (2013). Host specialization in ticks and transmission of tick-borne diseases: a review. Front Cell Infect Microbiol.

[99] Rappole JH, Hubálek Z (2003). Migratory birds and West Nile virus. J Appl Microbiol.

[100] Gubler DJ (2007). The continuing spread of West Nile virus in the western hemisphere. Clin Infect Dis.

[101] Levin II, Parker PG (2013). Comparative host-parasite population genetic structures: obligate fly ectoparasites on Galapagos seabirds. Parasitology.

[102] de Meeûs T, Béati L, Delaye C, Aeschlimann A, Renaud F (2002). Sex-biased genetic structure in the vector of Lyme disease, *Ixodes ricinus*. Evolution.

