# Supplementary figures and images for: Impact of life stage-dependent dispersal on the colonization dynamics of host patches by ticks and tick-borne infectious agents
Source: Parasit Vectors. 2017 Aug 4;10:375. doi: 10.1186/s13071-017-2261-y (PMC5544987; doi:10.1186/s13071-017-2261-y)

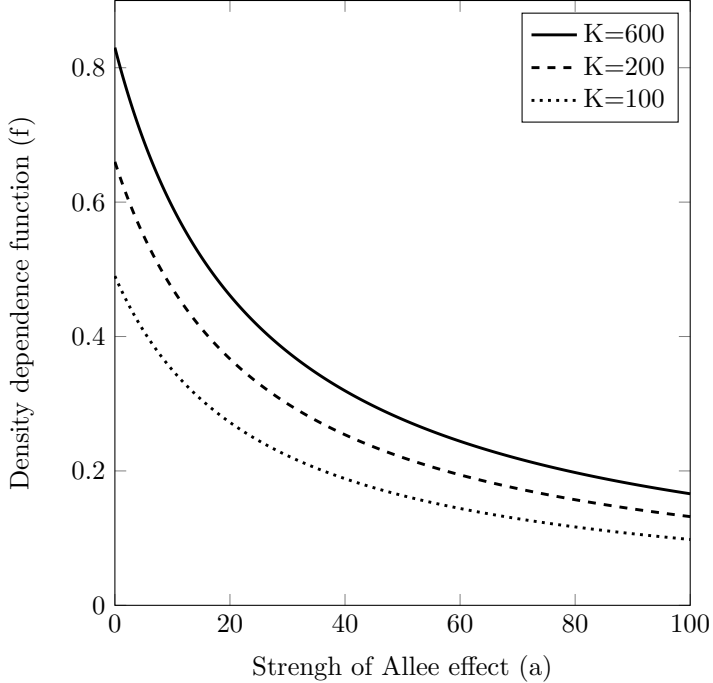

Supplement: Supplementary file 1 — Density-dependence function (f) with respect to varying strength of the Allee effect (a). Where K = 600, 200, 100 and a vary from 0 to 100. High a reduces the population reproductive function and represents notably the difficulty to locate a mate when the population density is low. (PDF 48 kb) [file 13071_2017_2261_MOESM1_ESM.pdf]

Infection prevalence in ticks

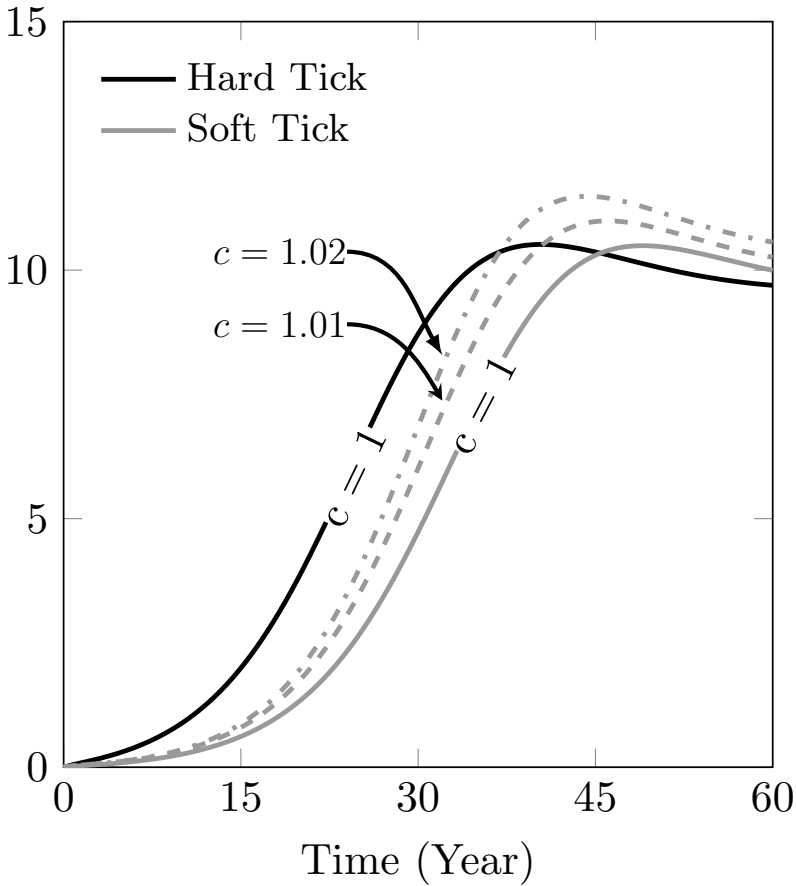

Supplement: Supplementary file 2 — To fully understand the consequences of the soft tick polyphasic cycle on vector-infection spread, we investigated the impact of an increase in the parameter feeding rate, c. This contact rate is only increased for nymphal and adult stages, as soft ticks take multiple short bloodmeals during those stages, while they only feed once during the larval stage, therefore c is kept equal to 1 at the larval stage. A slight increase in feeding rate leads to a rapid rise in the infection prevalence in soft tick systems, but as the soft ticks nidicolous lifestyle means that they probably feed on the same host or its offspring, soft tick multiple bloodmeal per life stage would certainly not increase vector-borne infection prevalence to the extent induced by direct changes of c. Figure S2. Vector-borne infection prevalence for different contact rates (i.e. tick feeding rate). Hard tick feed only once a year (c = 1 at all times), while soft can feed multiple times during nymphal and adult stages (c > 1 for nymphs and adults, c = 1 for larvae), which could potentially increase the opportunity for transmission, if they are to feed on different hosts. Default parameters are found in Table 1, θ = 0.001. Table S1. Parameters used for sensitivity analysis. (ZIP 217 kb) [file 13071_2017_2261_MOESM2_ESM.zip › DiseaseDynamics-contactrate-arrows.pdf]
